# Discovery of Glycoside Hydrolase Enzymes in an Avicel-Adapted Forest Soil Fungal Community by a Metatranscriptomic Approach

**DOI:** 10.1371/journal.pone.0055485

**Published:** 2013-02-05

**Authors:** Kazuto Takasaki, Takamasa Miura, Manabu Kanno, Hideyuki Tamaki, Satoshi Hanada, Yoichi Kamagata, Nobutada Kimura

**Affiliations:** Bioproduction Research Institute, National Institute of Advanced Industrial Science and Technology (AIST), Tsukuba, Ibaraki, Japan; Dowling College, United States of America

## Abstract

To discover the structural and functional novel glycoside hydrolase enzymes from soil fungal communities that decompose cellulosic biomass, transcripts of functional genes in a forest soil were analyzed. Pyrosequencing of the Avicel and wheat-amended soil cDNAs produced 56,084 putative protein-coding sequence (CDS) fragments, and the most dominant group of putative CDSs based on the taxonomic analysis was assigned to the domain *Eukarya*, which accounted for 99% of the total number of the putative CDSs. Of 9,449 eukaryotic CDSs whose functions could be categorized, approximately 40% of the putative CDSs corresponded to metabolism-related genes, including genes involved in carbohydrate, amino acid, and energy metabolism. Among the carbohydrate-metabolism genes, 129 sequences encoded glycoside hydrolase enzymes, with 47 sequences being putative cellulases belonging to 13 GH families. To characterize the function of glycoside hydrolase enzymes, we synthesized the putative CelA gene with codon optimization for heterologous expression in *Escherichia coli*, which was shown to be similar to the structure of plant expansins, and observed stimulation for cellulase activity on Avicel degradation. This study demonstrated that fungal communities adapt to Avicel and wheat decomposition and that metatranscriptomic sequence data can be reference data for identifying a novel gene.

## Introduction

The majority of prokaryotes living in the environment have not yet been cultured. Similarly, environmental community analysis based on 18S rRNA gene sequencing has shown that a wide variety of fungi and other eukaryotes exist in various environments and the majority of these species have also not been cultivated [1]–[3]. The diversity of the fungi and other eukaryotes together with their larger genome size compared to prokaryotes implies an enormous genetic and biological pool that can be explored to recover novel genes and enzymes, as well as entire metabolic pathways and their products [4].

Metatranscriptomics is a pioneering new field of mRNA-based functional community analyses [5]–[9]. It involves high-throughput detection and analysis of transcripts (RNA molecules) extracted from samples in which more than one microbial genome is present. Most notably, metatranscriptomic analysis based on expressed genes is a more suitable means to unravel eukaryotic community functions in the environment [5]–[7] in an ecological context, because metagenomic analysis based on DNAs cannot determine the structural genes whose introns are excluded, let alone detect ecologically relevant active functions. An RNA-based metatranscriptomic approach can circumvent the recurrent problems in the conventional metagenomic approach, and 3′ poly-A tails-specific purification and subsequent reverse transcription lead to construction of a cDNA library, allowing comprehensive analyses of the eukaryotic genes specifically expressed.

Cellulose is the most abundant biopolymer in nature, estimated to account for about 1.5×10^12^ tons of the total annual biomass production [10]. Cellulosic biomass from agricultural crop residues, grasses and wood represents an abundant renewable resource that is becoming increasingly important as a future source of energy. Fungi are the main sources of cellulases and hemicellulases, enzymes that are used to degrade the cellulosic biomass to simple sugars [11], [12]. The degradation of cellulose by fungal microbes plays an important role in recycling cellulose in the biosphere [13].

Here, we attempted to explore the genetic and functional diversity of eukaryotes by applying a transcriptomic approach to target genes encoding glycoside hydrolase enzymes. We amended the soil sample with cellulose and wheat bran and incubated it as a soil suspension, anticipating that the responsible genes would be induced, expressed and upregulated. To investigate the taxonomic diversity of the fungal community in Avicel and wheat bran-amended soil, the clone libraries of 18S ribosomal genes were constructed and sequenced. Novel genes encoding glycoside hydrolase enzymes were identified from metatranscriptomic sequence data, and synthesized and expressed to yield the active enzymes.

## Results

### Taxonomic Diversity of the Soil Eukaryotic Community

**Figure 1:**
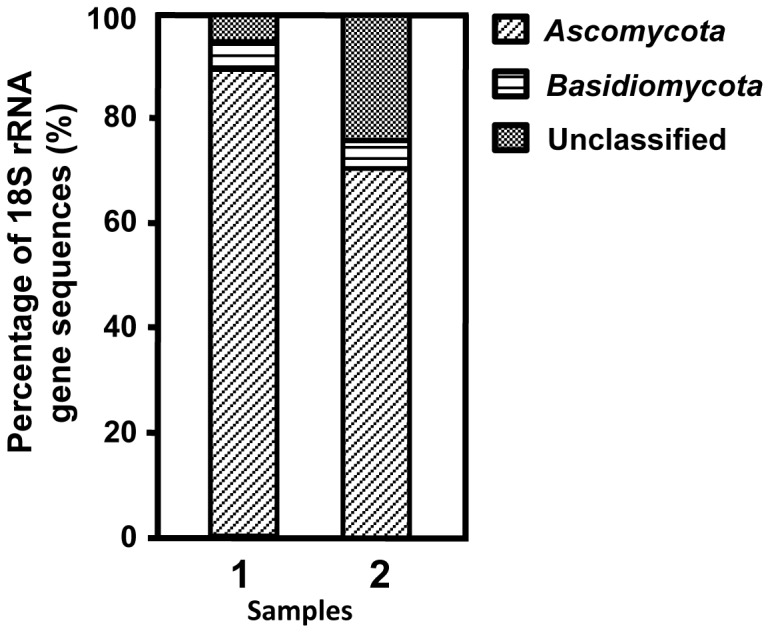
Taxonomic classifications of the partial 18S rDNA sequences amplified from the amended soil DNA (1) and the unamended soil DNA (2). Microbe classifications: diagonal lines, *Ascomycota*; horizontal lines, *Basidiomycota*; dotted area, unclassified fungi. Unclassified sequences could not be clearly attributed to known eukaryotic taxonomic groups.

To examine the effect of Avicel/wheat bran amendment on the transition of fungal community composition, the 18S rRNA genes from the amended soil were amplified using fungi-specific PCR primers, and an 18S rRNA gene-based community analysis was performed. A clone library was constructed from PCR products amplified from the Avicel/wheat bran-amended soil DNA as a template. As a comparison, a clone library was also constructed for the unamended soil suspension after the same period of incubation. Ninety-six clones from each library were screened and sequenced. The sequence analysis grouped the clones into 16 distinct types [operational taxonomic units (OTUs)] in the amended soil, and 19 types of OTUs in the unamended soil (i.e., sequences with greater than 99% similarity were regarded identical) (Figure S2). A total of 96 clones from the 18S rRNA gene library of the amended soil were analyzed in order to estimate the diversity of the fungal community. Phylogenetic analysis revealed that the clone sequences from the amended soil were affiliated with at least two classes of the domain *Eukarya.* The most dominant group was allocated to the phylum *Ascomycota*, accounting for 88% of the total number of clones (Figure 1). The second most dominant group of the clone library, represented by six clones (6%), was classified into the phylum *Basidiomycota* group. The major groups in the unamended soil library were the *Ascomycota* (67%) and the *Basidiomycota* (6%).

### Total RNA Extraction and cDNA Synthesis

Extractions were performed on 10 g of the soil sample collected from soil slurry, and a total of 1.17 mg of environmental RNA was recovered (yield of ca. 0.120 mg/g of soil).

Capillary electrophoresis of the extracted total RNA yielded four major peaks (Figure S1a), in which the last three peaks were comprised mainly of prokaryotic and eukaryotic rRNAs, while the first large peak corresponded to small debris such as partially digested RNA.

The extract was enriched in eukaryotic polyadenylated mRNA based on affinity capture on columns coated with poly-dT and 9.21 µg of mRNA were recovered ( = 0.79% of the total extracted RNA). Capillary electrophoresis of an aliquot sample (Figure S1b) showed significant removal of rRNAs and small debris. Finally, the purified extract (100 ng of mRNA) was used for the synthesis of cDNAs ranging in size from 200 bp to more than 2 kb (Figure S1c).

### cDNA Sequencing and Sequence Analysis

The sequence features are shown in Table 1. A full sequencing run with the GS-FLX sequencer yielded 93,415,467 bases from 400,465 reads (average length: 232 bases). After trimming and assembly, 17,195 contigs and 39,598 singlets were yielded. These sequences were assigned by BLASTX against the non-redundant (nr) database at NCBI with an e-value <10^−8^. As a result, 56,084 CDSs were predicted: 44% of the sequences yielded no positive hits in the database, and were therefore considered new hypothetical proteins, 34% of the sequences were homologous to genes coding protein sequences of unknown function (conserved hypothetical proteins), and the remaining 22% of the sequences corresponded to genes coding protein sequences of known function.

**Table 1:** Sequence features of the metatranscriptomic analysis from the soil.

Characteristic	Value
Total Size (bp)	93,415,467
Total number of reads	400,465
Average read length (bp)	232
Average GC content (%)	52.4
Number of contigs	17,195
Number of singlets	39,598
Putative protein-coding sequences	56,084
Functionally assigned	12,138
Conserved hypothetical protein	19,273
Hypothetical protein	24,673

**Figure 2:**
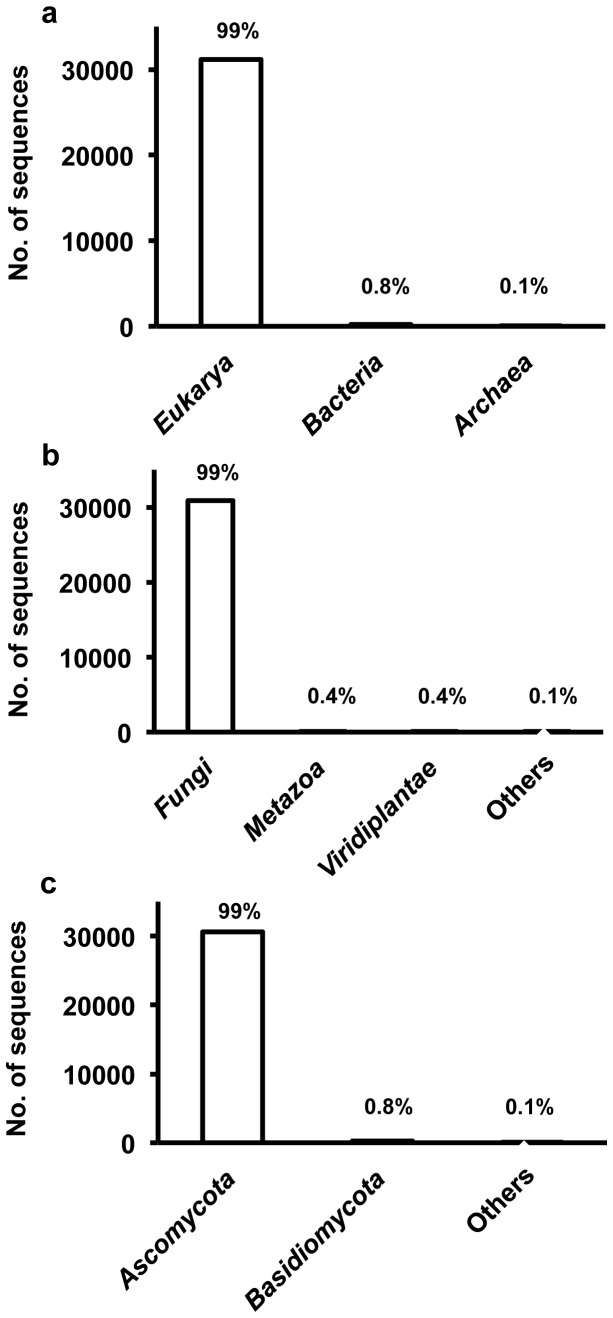
Taxonomic classifications of the sequenced cDNAs based on BLAST analysis. Sequences that did not return any significant hits (usually E values greater than 10^−8^) were classified as unknown. The figure indicates the Domain (a), Kingdom (b) and Phylum levels (c).

The putative taxonomic origins of the 56,084 synthesized cDNA sequences were determined using a BLAST search according to the NCBI taxonomic hierarchy (Figure 2). ased on the taxonomic analysis, the most dominant group of putative CDSs was assigned to the domain *Eukarya*, which accounted for 99% of the total number of putative CDSs. The other groups of putative CDSs were determined to be, in order of abundance, in the domains *Bacteria* and *Archaea* (0.8% and <0.1%, respectively). Most of the sequences (99%) assigned to the domain *Eukarya* were classified into the kingdom *Fungi*. The other groups of putative CDSs assigned to the domain *Eukarya* were determined to be, in order of abundance, in the kingdoms *Metazoa* and *Viridiplantae* (0.4% and 0.4%). Phylogenetic analysis of the fungal sequences revealed that the main taxonomic group in the *Fungi* was the phylum *Ascomycota* (99%). Another group of putative CDSs assigned to the kingdom *Fungi* was determined to be in the phylum *Basidiomycota* (0.8%). The remaining 0.1% of the putative CDSs assigned to the kingdom *Fungi* could not be clearly classified into a known taxonomic group.

**Figure 3:**
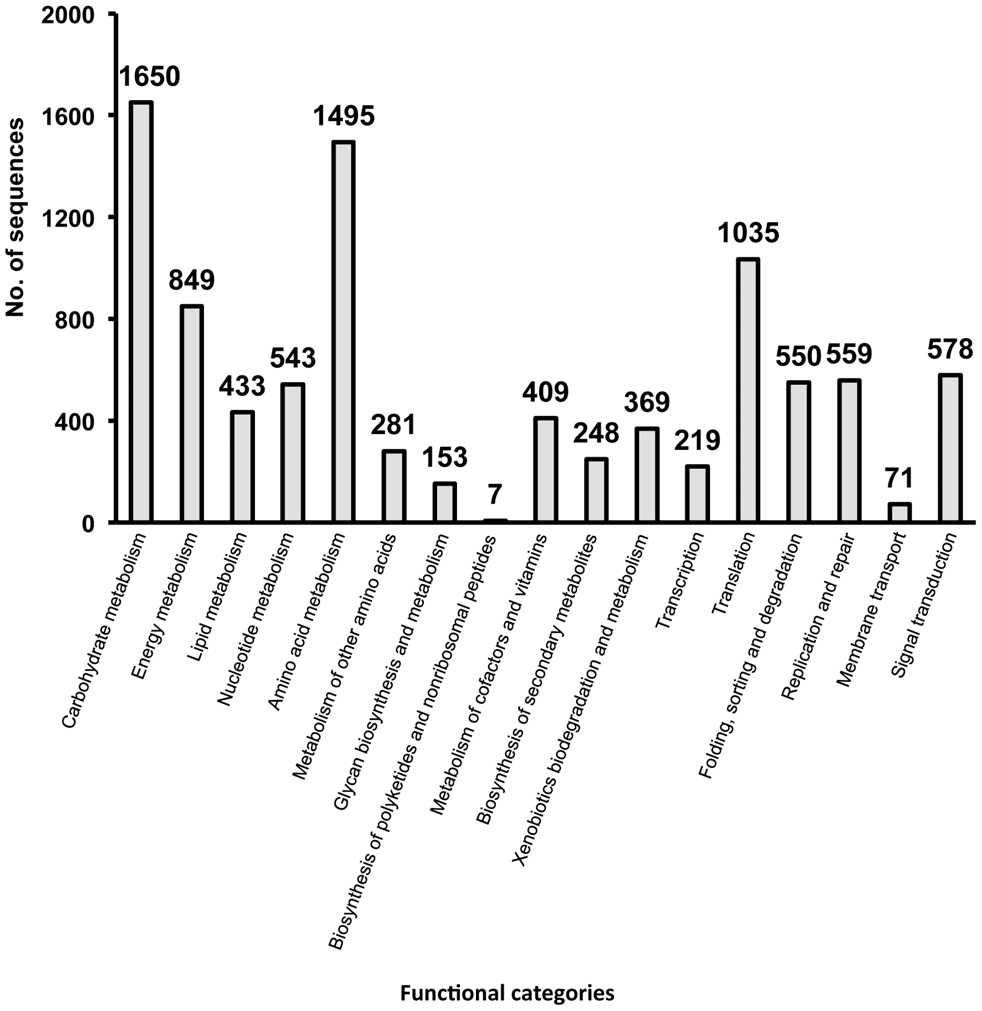
Functional classifications of the putative CDSs derived from the metatranscriptomic approach. The functional genes were determined using a BLASTP search according to the KEGG database (http://www.kegg.com/kegg/) with expected values above 1×10^−8^. Multifunctional genes were classified in redundant categories. Functional categories: carbohydrate metabolism; energy metabolism; lipid metabolism; nucleotide metabolism; amino acid metabolism; metabolism of other amino acids; glycan biosynthesis and metabolism; biosynthesis of polyketides and nonribosomal peptides; metabolism of cofactors and vitamins; biosynthesis of secondary metabolites; xenobiotics biodegradation and metabolism; transcription; translation; folding, sorting and degradation; replication and repair; membrane transport; and signal transduction.

The predicted 31,411 CDSs were assigned to functional categories by the KEGG Orthology database with an e-value <10^−8^, resulting in 9,449 sequences corresponding to genes coding proteins of known function (Figure 3). Approximately 40% of the sequences corresponded to metabolism-related genes involved in carbohydrate (category A), amino acid (category E) and energy metabolism (category B). Housekeeping genes involved in translation (category M) and replication and repair (category Q) were also abundant. On the other hand, the number of sequences classified into the functional categories of membrane transport (category P) and biosynthesis of polyketides and nonribosomal peptides (category H) were far fewer.

### Profiling of the Genes Coding Biomass-catalysts

**Figure 4:**
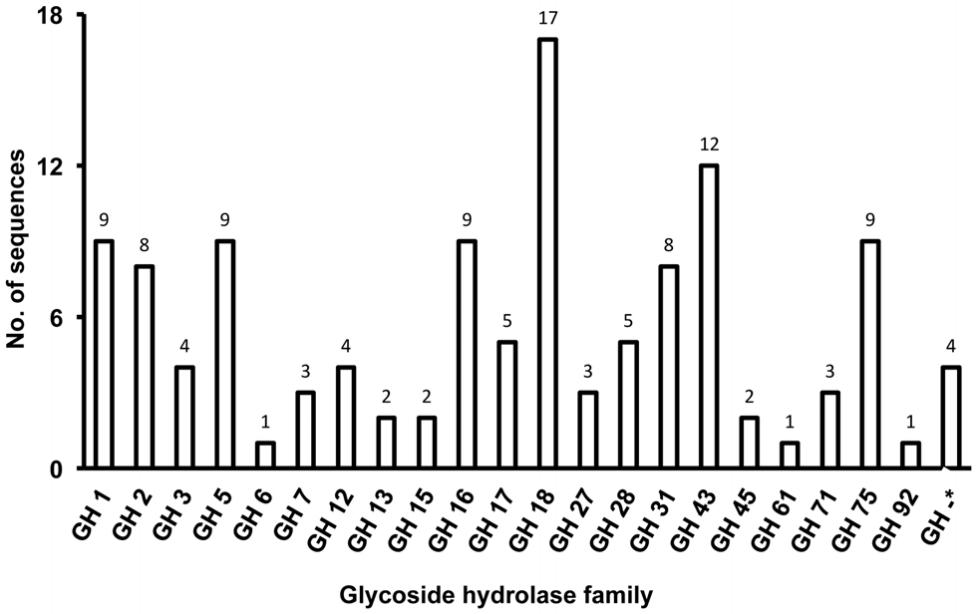
Classifications of putative glycoside hydrolases (GH) from metatranscriptomic information. Uniplot KB (Wu et al., 2006) and CAZy databases (Cantarel et al., 2009) were used to confirm sequence-based similarities of glycoside hydrolases. Stars (*) indicate sequences unclassified in GH families.

We identified 129 sequences encoding glycoside hydrolase enzymes from predicted CDSs based on the information from the BLAST and motif search (Figure 4 and Table S1). The sequences coding glycoside hydrolase enzymes were classified into the 22 members of the Glycoside Hydrolase (GH) family. The most dominant was the GH18 family, accounting for 14% (17 sequences) of the total number of sequences coding glycoside hydrolase enzymes. The other groups to which sequences belonged were, in order of abundance, the GH43, GH1, GH5, GH16, and GH75 families (10%, 7%, 7%, 7%, and 7%, respectively). Genes encoding cellulase, such as glucanase, cellobiohydrolase and β-glucosidase, were classified into various GH families, while almost all of the genes encoding hemicellulase, such as xylanase and xylosidase, were classified into GH43, excluding FX024519 (GH16) and FX042773 (GH3). These results indicated that the soil eukaryotic communities have the metabolic potential to hydrolyze carbohydrate components of the plant cell wall, including cellulose, xylan components such as acetylxylan, arabinosides, and xylosides, and fructosides, galactosides, glucosides, and mannosides.

One putative swollenin and two putative expansins were also identified from the metatranscriptomic sequence data (Figure 4a and Table S1). FX035782 was similar to swollenin of *Trichoderma asperellum* (97.7% identity), while two putative expansins (FX003685 and FX036114) exhibited 79.1% identity with extracellular cellulase of *Neosartorya fischeri* NRRL 181 and 67.5% identity with extracellular cellulase of *Aspergillus fumigatus* Af293, respectively. Expansin and swollenin are involved in loosening and swelling of plant cell walls and cellulose fiber by disrupting non-covalent bonding between cellulose microfibrils and matrix glucans [14], but they are rarely found proteins.

### Characterization of a Novel Expansin Enzyme Expressed in *E. coli* Cells

The amino acid sequence of a protein encoding FX003685 has 79% similarity with extracellular cellulase (CelA) from *Neosartorya fischeri* (XP_001259883). The deduced amino acid sequence of the FX003685 protein has the typical polypeptide organization conserved among the members of the expansin family. The DNA fragment encoding a putative extracellular cellulase CelA protein of *Neosartorya fischeri* NRRL 181 was chemically synthesized, which showed the highest amino acid sequence similarity to the sequence (FX003685) in metatranscriptomic sequence data. Plasmid pEX19 containing the CelA gene was constructed by using expression vector pET19b, and then the plasmid was transfected into *E. coli* strain BL21 (DE3) and Rosetta2 (DE3) cells. A clone was IPTG-induced and the crude cell extracts and purified protein (His-tagged) were subjected to sodium dodecyl sulfate-polyacrylamide gel electrophoresis analysis. A distinctive overexpressed protein with a molecular mass of approximately 36 kDa was observed in *E. coli* harboring pEX19. These results agreed with the theoretical molecular mass of a CelA protein that encodes 352 amino acid sequences (36.2 kDa).

**Figure 5:**
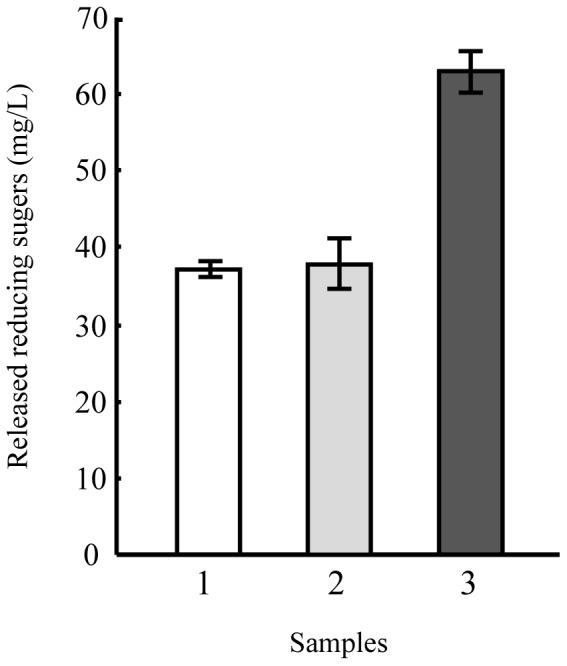
Synergism of cellulase in the presence of the CelA enzyme. The reaction mixture (200 µl final volume) contained 0–1 mg Accellerase 1500 protein per g of Avicel, 1.0% (w/v) Avicel, and 0–10 mg CelA protein per g of Avicel in 0.05M acetate buffer (pH 5.0). The mixture was shaken at 50°C for 1 h before quantification of reducing sugars; sample 1, cell lysate of the *E. coli* BL21 cells harboring plasmid pET28, sample 2, cell lysate of the *E. coli* BL21 cells producing recombinant FX003685 enzyme.

In order to characterize the function of the CelA gene product, the enzyme assay of CelA with *E. coli* harboring pMQ19 was done by measuring the glycoside hydrolase activity of Avicel as described in the Materials and Methods. Figure 5 shows the enzymatic hydrolysis of Avicel by cellulase alone, CelA alone or a combination of cellulase and CelA. Incubation of Avicel with CelA alone did not result in a significant amount of reducing sugar, which is similar to the results of previous studies which found that plant expansins possess no hydrolytic activity [15]–[17]. When the Avicel was incubated with cellulase alone, the extent of sugar production over time was small due to the low level of cellulase loading (1.6 FPU/g cellulose). However, when the Avicel was incubated with a mixture of CelA (10 mg per g of cellulose) and cellulase for 1 h, the reducing sugar yield from the Avicel was 1.8-fold greater than that of the control containing Avicel and cellulase alone.

## Discussion

In the present study, the glycoside hydrolase enzymes in an amended soil sample were identified by using a metatranscriptomic dataset. Remarkably, the genes encoding glycoside hydrolase enzymes such as glucanase, β-glucosidase, α-glucosidase, and chitinase were identified in the identifiable cDNA sequences from amended soil. The glucanases belonging to the families endo-1, 3-β-glucanase GH16, endo-β-1, 4-glucanase GH5, and endoglucanase GH12 were particularly abundant in the amended soil. Endoglucanase breaks internal bonds to disrupt the crystalline structure of cellulose and expose individual cellulose polysaccharide chains. Most of the β-glucosidase in the amended soil belonged to family GH1. The β-glucosidase family GH1 hydrolyzes terminal, non-reducing β-D -glucosyl residues with release of β-D-glucose. These results indicated that the glucanase and β-glucosidase identified in the metatranscriptomic dataset may have played a role in cellulose degradation in the amended soil. Fungal chitinases are important for utilization of chitin as carbon and as an energy source in soil, and for the apical growth of hypha, hyphal branching and cell separation, e.g., release of spores [18].

Furthermore, we identified one putative swollenin and two putative expansins from the functional analysis of predicted CDSs using the metatranscriptomic sequence data. Swollenin, a protein first characterized in the saprophytic fungus *Trichoderma reesei*, contains an N-terminal carbohydrate-binding module family 1 domain (CBD) with cellulose-binding function and a C-terminal expansin-like domain [14]. Expansins are a family of closely-related nonenzymatic proteins found in the plant cell wall, bacteria, and fungi, which cause wall stress relaxation and irreversible wall extension [19], [20]. Thus the metatranscriptome analysis allowed us to detect sequences associated with particular environmental conditions that may not be as readily identified in function-based metagenomic studies. On the other hand, 35 sequences showed low amino acid sequence similarity (less than 70%) to any known glycoside hydrolase enzyme-coding sequences. Moreover, a number of the CDS sequences from amended soil had no homology to any sequences in the GenBank databases. Therefore, unknown functional genes enabling cellulose degradation may also exist in the amended soil.

We demonstrated that enrichment of the microbial populations in the presence of cellulose biomass led to changes in the fungal community. From microbial community analysis based on the 18S rRNA gene sequence, a difference was found between the two 18S rRNA gene clone libraries. Members of *Ascomycota* were dominant in the amended soil library, accounting for 88% of the 18S rRNA gene in the amended soil library. Among the isolated microbes with the ability to degrade cellulose, most cellulose-degrading fungi belong to the phylum *Ascomycota,* which includes species such as *Trichoderma reesei, T. harzianum,* and *Aspergillus niger* [21]. The population of the *Ascomycota* was elevated in the enriched sample, and may play an important role in cellulose degradation in the environment.

Recently, new sequencing platforms, such as 454 pyrosequencing, have allowed the metatranscriptome analysis of complex microbial communities [6], [7], [9], [22]. However, the inherent instability of RNA molecules has been one of the most limiting factors for the development of metatranscriptomics. Even though extensive efforts have been made to develop methods for extracting RNA from numerous environmental communities, it remains challenging to establish protocols to easily extract and purify eukaryotic RNA from a complex environmental matrix such as soil, due to the coextraction of humic acids and other organic compounds from the soil sample (Table 2) [8], [23]–[25]. Here, we present an experimental and analytical approach that extracted mRNA from an amended soil sample.

**Table 2:** Characteristics of soil used in this study.

Characteristics	Forest soil
pH [H_2_O]	4.9
pH [KCl]	4.2
Electric conductivity (mS/m)	5.4
Total carbon (%)	11
Total nitrogen (%)	0.68
Total humic substance (%)	19
Total phosphorus content (mg/kg)	1,100
Available phosphorus content (mg/kb)	2.0
Exchangeable cations	
K (cmol (+)/kg)	0.48
Mg (cmol (+)/kg)	1.0
Ca (cmol (+)/kg)	3.8
Na (cmol (+)/kg)	0.096
Mn (cmol (+)/kg)	0.031
Al (cmol (+)/kg)	0.96

Access to the entire metagenome of these as-yet-uncultured organisms would provide a completely new gene pool, which could yield novel enzymes and proteins of potential industrial or medical use [26]. It is now common practice to isolate DNA directly from environmental samples and construct DNA libraries to access the metagenome. Through the function-based screening method of the metagenomic approach, glycoside hydrolases were cloned from the microbial consortia in a treated soil culture and rumen metagenome [27], [28]. Also, glycoside hydrolase genes were cloned from a switchgrass-adapted compost community by the sequence-based screening method of the metagenomic approach [29]. However, fungal genetic information still remains an unknown frontier in the analysis of environmental resources. In the present work, metatranscriptomic approaches provided unprecedented access to the genetic information of fungi with respect to biomass conversion processes. It can be anticipated that the combination of microbial community analysis and metatranscriptome analysis will lead to the discovery of new biomass-conversion strains and novel biomass-catalytic genes from environmental samples. Cloning of the gene fragments encoding glycoside hydrolase enzymes is currently under way to obtain more information on these enzymes. These approaches may provide a greater understanding of the functions and diversity of carbohydrate metabolic enzymes and could promote the application of these enzymes to the decomposition of cellulosic biomass.

## Materials and Methods

### Soil Sampling

About 200 g of humic soil was collected from a forest at the National Institute of Advanced Industrial Science and Technology (AIST; Tsukuba city, Japan), located at 36° 3′ 48.7614′′N and 140° 7′ 46.9878′′E, on October 20, 2008 (soil temperature, 15°C at 5 cm depth). The soil sample was sieved (2 mm mesh size) to remove fine roots, leaves and other organic debris. After sampling, the soil was immediately used for the following step without frozen storage. The characteristics of soil used in this study are shown in Table 2. The substratum was a nutrient-poor, non-calcareous sand (*ca*. 98–99% sand, pH 5.5) with *ca*. 0.5% of organic matter.

### Enrichment of Soil Biomass

Soil suspensions were prepared from the soil sample by mixing 100 g of the soil and 1 L of distilled water containing 1 g of wheat bran and 1 g of microcrystalline cellulose (Avicel, Fluka Biochemika, Buchs, Switzerland). A soil suspension unamended with Avicel and wheat bran was used as a control. Erlenmeyer flasks, each containing 5 liters of soil suspension, were gently shaken with a rotary shaker under aerobic conditions for 6 days at room temperature. After incubation, the samples were collected by centrifugation (10,000×g, 30 min) and were used for subsequent DNA and RNA extraction.

### RNA and cDNA Preparation

Soil slurry samples were collected by centrifugation at 10,000×g for 15 min. Total RNA extraction from 10 g of the collected soil samples was performed by using a FastRNA Pro Soil-Direct Kit (Qbiogene, Solon, OH) according to the manufacturer’s instructions. A DNase-treatment was conducted to remove genomic DNA from total RNA by using Recombinant DNaseI (RNase-free) (TaKaRa, Kyoto, Japan). After ethanol precipitation, the purified total RNA was stored at −80°C until the subsequent step. Polyadenylated eukaryotic mRNA was purified by affinity capture on Sephadex coated with poly-dT oligonucleotides as described in the Oligo (dT) kit (TaKaRa, Kyoto, Japan) instruction manual. An RNeasy MiniElute Cleanup Kit (QIAGEN, Valencia, USA) was used to remove small molecular debris. RNA purity and concentrations were estimated by a Nano Drop spectrophotometer (Thermo Scientific, Yokohama, Japan). RNA quality and quantity were estimated by a Bioanalyzer (Agilent, Tokyo, Japan).

Synthesis of cDNA from the purified mRNA was performed using a cDNA Synthesis Kit (TaKaRa). First, the poly (A) RNA was reverse-transcribed with an Oligo(dT)-T7 primer containing a T7 promoter sequence, and then the double-stranded cDNA was synthesized. Next, the cDNA templates were transcribed *in vitro* with T7 RNA polymerase (TaKaRa), yielding large amounts of antisense RNA (aRNA). Finally, aRNA was further reverse-transcribed to cDNA with biotinated oligo (dT) primer for pyrosequencing.

### cDNA Sequencing and Sequence Analysis

Sequencing of the synthesized cDNA was performed by a Genome Sequencer FLX system (Roche, Tokyo, Japan). Trimming of low-quality sequences and assembly were performed by the Newbler software package (Roche) with a default value. Sequences were compared with the NCBI-nr database (http://www.ncbi.nlm.nih.gov/) at the National Center for Biotechnology Information (NCBI) using BLASTX with an e-value <10^−8^. Top hits with bit scores over 50 were assigned protein-coding sequences (CDSs). Nucleotide sequences were deposited in the EMBL/GenBank/DDBJ database under accession numbers FX000001 to FX056084.

The global functions of the CDSs were determined using a BLAST search according to the KEGG database (http://www.kegg.com/) with an e-value <10^−8^. KEGG Orthology **(**KO) (http://www.kegg.com/kegg/ko.html) categories were used for functional classification of the CDSs [30]. Multifunctional sequences were classified in redundant categories. Sequences associated with carbohydrate catabolism were referred to the UniprotKB (http://www.expasy.ch/) [31] and classified into the Glycoside Hydrolase (GH) family according to the CAZy databases (http://www.cazy.org/) [12].

### Cloning Analysis of the 18S rRNA Genes

The total DNAs from the soil samples with and without substrate amendment were extracted by using a Fast DNA Spin Kit for Soil (Qbiogene, Irvine, CA) according to the manufacturer’s instructions. The fungal 18S rRNA genes were amplified by PCR from soil DNA using the primers NS1 and MF3 [32]. PCR amplification was performed with a GeneAmp PCR System 9700 (Applied Biosystem, Tokyo, Japan). The PCR mixture contained 100 ng of environmental DNA, 200 nM of each primer, 1 mM MgCl_2_, 200 uM of each dNTP, 1U of *Taq* polymerase and an appropriate buffer (TaKaRa), in a final volume of 100 µl. PCR was performed under the following conditions: 95°C for 2 min, followed by 20 cycles of 95°C for 10 s, 50°C for 15 s, 72°C for 2 min, and a final extension step at 72°C for 5 min. PCR products were purified with a Sephadex S-400 spin column (GE Healthcare, Tokyo, Japan), and a TOPO TA cloning kit for Sequencing (Invitrogen, Tokyo, Japan) was used for the cloning of the 18S rRNA genes according to the manufacturer’s instructions. The clone sequencing was performed by a CEQ™ 2000 XL DNA analysis system (Beckman Coulter, Tokyo, Japan). Nucleotide sequences were deposited in the EMBL/GenBank/DDBJ database under accession numbers AB548995 to AB549186.

The sequenced 18S rRNA genes were manually corrected and edited. A BLAST search was performed against the non-redundant (nr) database at NCBI (http://www.ncbi.nlm.nih.gov/) [33]. The 18S rRNA genes were screened for putative chimeras using the ChimeraCheck program at the Ribosomal Database Project II (http://rdp8.cme.msu.edu/html/index.html) [34]. Rarefaction curves for the 18S rRNA gene sequences were calculated using Analytical Rarefaction version 1.3 software (http://www.huntmountainsoftware.com/).

### Cloning and Expression of the CelA Gene

The CelA gene was amplified by PCR with the primers 5′- CAGCATATGTCCCCTATGGTCGGT -3′ (*Nde*I site underlined) for Exf2, and 5′- GGATCCTTAGAGGTTAGACTTAGCG-3′ (*Bam*HI site underlined) for Exr2. The chemically synthesized DNA fragment encoding a protein of *Neosartorya fischeri* NRRL 181 (XP_001259883) was used as a template, which showed the highest amino acid sequence similarity to the sequence (FX003685) identified from metatranscriptomic data. The PCR product was digested by restriction enzymes and cloned into the pET19b expression vector (Novagen). The resulting plasmid, pEX19, was transfected into *E. coli* BL21 (DE3) and Rosetta2 (DE3) cells (Novagen).

The cultures were incubated at 37°C for 3 h with shaking at 200 rpm, followed by the addition of IPTG to 1.0 mM and subsequent incubation at 28°C for 15 h to induce protein expression. The culture media of *E. coli* were centrifuged at 10,000 rpm for 10 min and resuspended in phosphate buffer (pH 6.8). Cell breakage was carried out until the solution was cleared by using an ultrasonic disintegrator (Sonicator BRANSON sonifer 250), and the resulting solutions were centrifuged at 7,000 rpm for 10 min. The recombinant proteins were purified from the soluble fraction by HIS-Select Nickel Affinity Gel (SIGMA-ALDRICH) chromatography according to the manufacture’s instruction.

### Sodium Dodecyl Sulfate-polyacrylamide Gel Electrophoresis (SDS-PAGE)

Crude cell extract and His-tagged crude cell extract were treated with marker dye (1% SDS, 1% 2-mercaptoethanol, 10 mM Tris-HCl (pH 6.8), 20% glycerin, 1 mg ml^−1^ bromphenol blue), and heated for 5 min at 98°C. For SDS-PAGE analysis, the resulting samples were subjected to 10% PAGE gel under 20 mA for 40 min by using 10× SDS-PAGE running buffer (1% SDS, 3% Tris, 14.4% glycin). Proteins were detected by staining with a Bio-Safe™ Coomassie G-250 stain (BIO-RAD).

### Enzymatic Assay of the CelA Protein

To evaluate the synergistic effect on cellulose hydrolysis between cellulase and the CelA enzyme, which has generally been observed in expansin-like proteins [35], the amount of reducing sugar released from the Avicel was determined after treatment with expansin protein and cellulase as described previously [36]. The expansin protein derived from the CelA gene and a commercial cellulose mixture (Accellerase 1500; Genencor International, Rochester, NY) were diluted to a final concentration of 0–10 mg protein and 0–1 mg protein per g of Avicel, respectively, in 200 µl final volume of 1.0% (w/v) Avicel suspension with 0.05 M acetate buffer (pH 5.0). The reaction mixture was incubated for 1 h at 50°C. The reducing sugar levels were evaluated by the Nelson-Somogyi method [37] and the absorbance at 595 nm was then measured. The values are the means ± standard deviations of three independent measurements.

## Supporting Information

Figure S1 Soil RNA extraction from the amended soil sample and conversion of mRNA into complementary DNAs. Capillary electrophoresis profiles of total RNA extracted from the humic soil (a) and of RNA obtained after affinity capture on oligo-dT and a cleanup column for removal of small debris (b). The double-stranded cDNAs obtained by PCR amplification of the reverse-transcribed mRNAs ranged in size from ca. 200 bp to more than 2 kb (c).
(TIF)

Figure S2 Phylogenetic tree based on the 18S rRNA sequences in this study. The sequences are aligned, and the base tree was constructed with >1000 nt sequences by the neighbor-joining method. Bold types indicate the clones obtained in this study, and those labeled with closed squares (▪) and open squares (□) were derived from the treated soil and the untreated soil, respectively. The 16S rRNA sequence of *Escherichia coli* (CP001396) was used as an outgroup to root the tree. Closed circles (•) at nodes indicate branches with a bootstrap value of >85%.
(TIF)

Table S1 List of putative lignocellulolytic enzymes identified in the metatranscriptomic data.
(TIFF)
