# Supplementary figures and images for: Discovery of Glycoside Hydrolase Enzymes in an Avicel-Adapted Forest Soil Fungal Community by a Metatranscriptomic Approach
Source: PLoS One. 2013 Feb 5;8(2):e55485. doi: 10.1371/journal.pone.0055485 (PMC3564753; doi:10.1371/journal.pone.0055485)

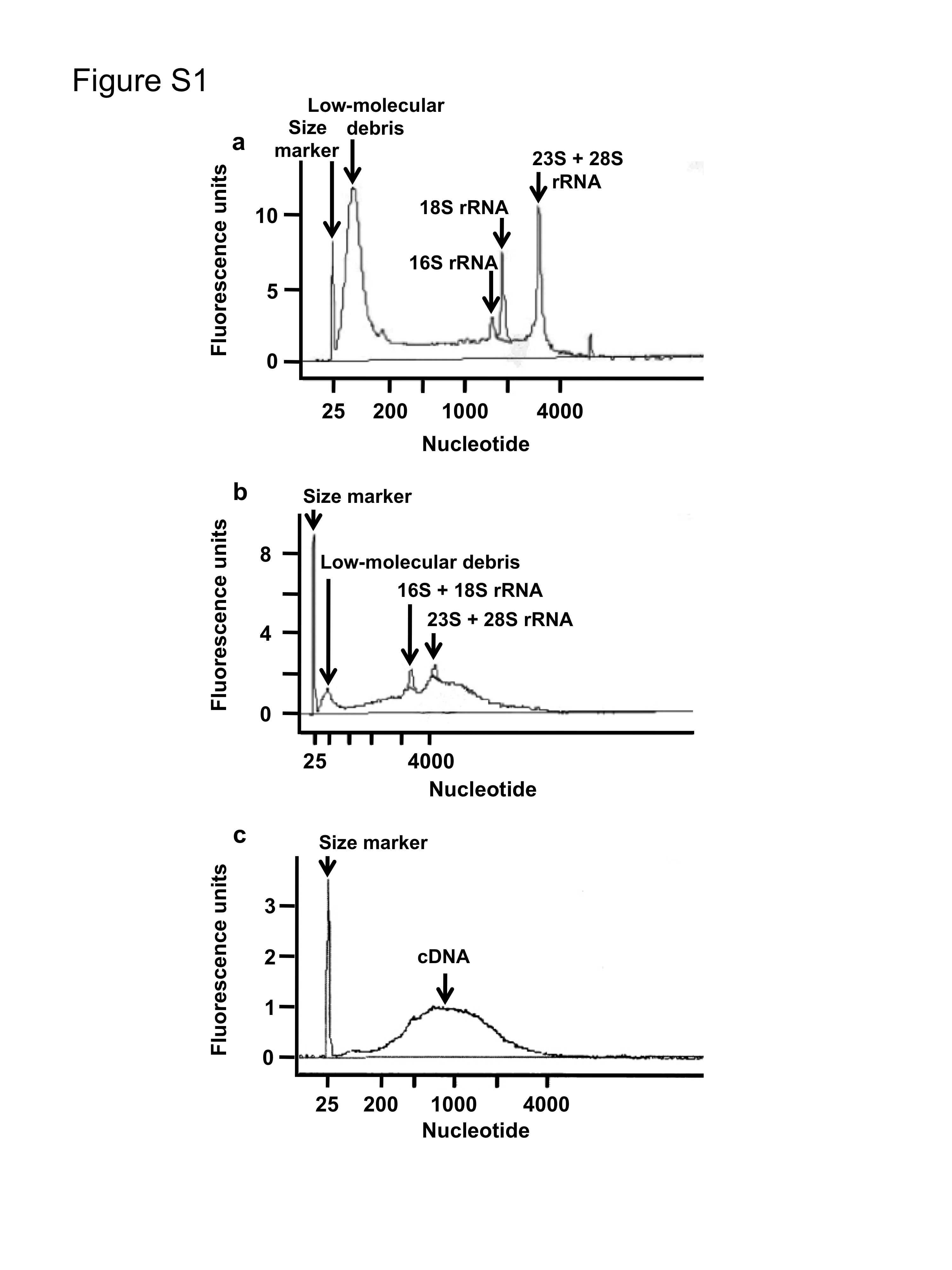

Supplement: Figure S1 — Soil RNA extraction from the amended soil sample and conversion of mRNA into complementary DNAs. Capillary electrophoresis profiles of total RNA extracted from the humic soil (a) and of RNA obtained after affinity capture on oligo-dT and a cleanup column for removal of small debris (b). The double-stranded cDNAs obtained by PCR amplification of the reverse-transcribed mRNAs ranged in size from ca. 200 bp to more than 2 kb (c). (TIF) [file pone.0055485.s001.tif]

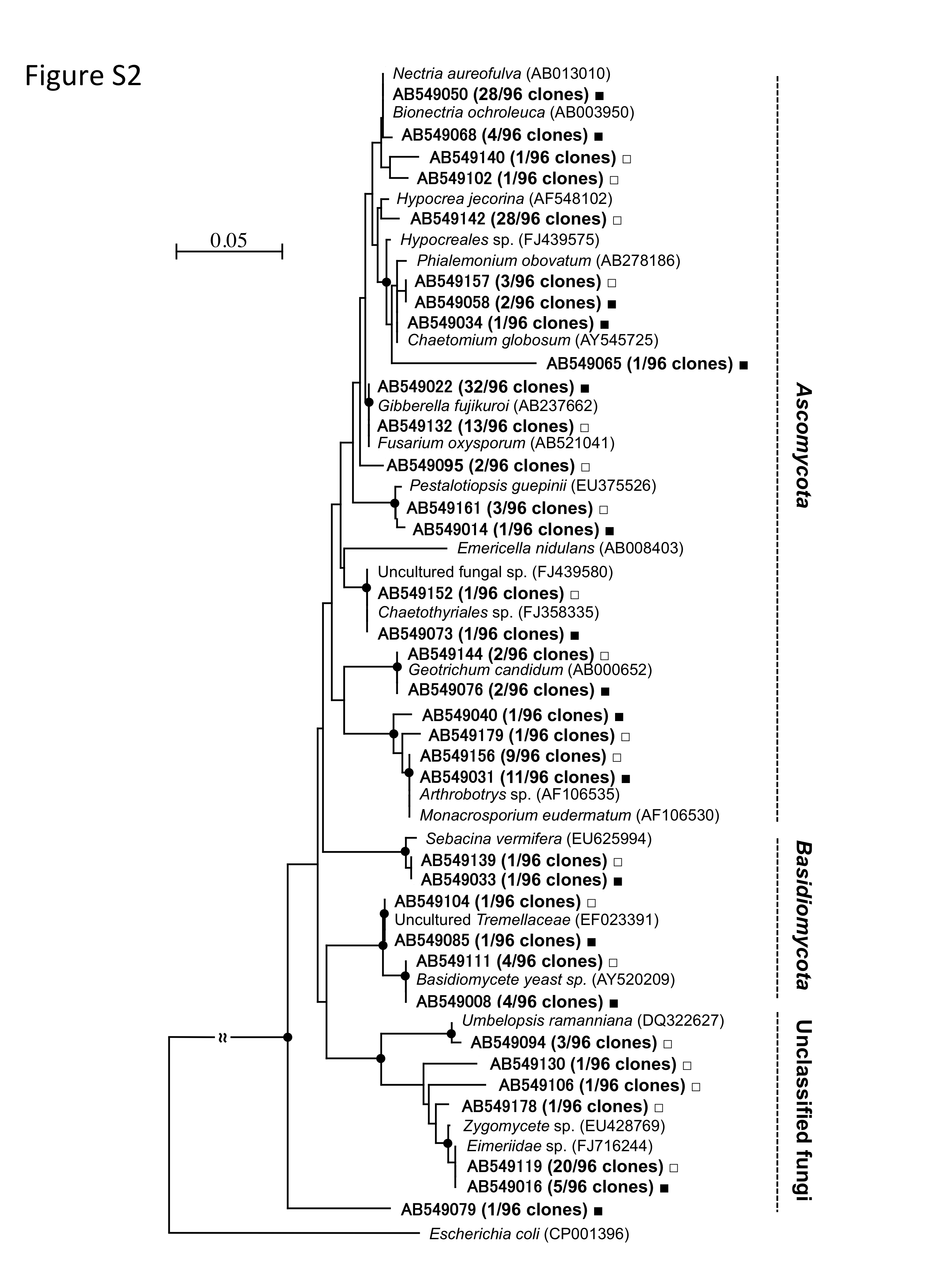

Supplement: Figure S2 — Phylogenetic tree based on the 18S rRNA sequences in this study. The sequences are aligned, and the base tree was constructed with >1000 nt sequences by the neighbor-joining method. Bold types indicate the clones obtained in this study, and those labeled with closed squares (▪) and open squares (□) were derived from the treated soil and the untreated soil, respectively. The 16S rRNA sequence of Escherichia coli (CP001396) was used as an outgroup to root the tree. Closed circles (•) at nodes indicate branches with a bootstrap value of >85%. (TIF) [file pone.0055485.s002.tif]

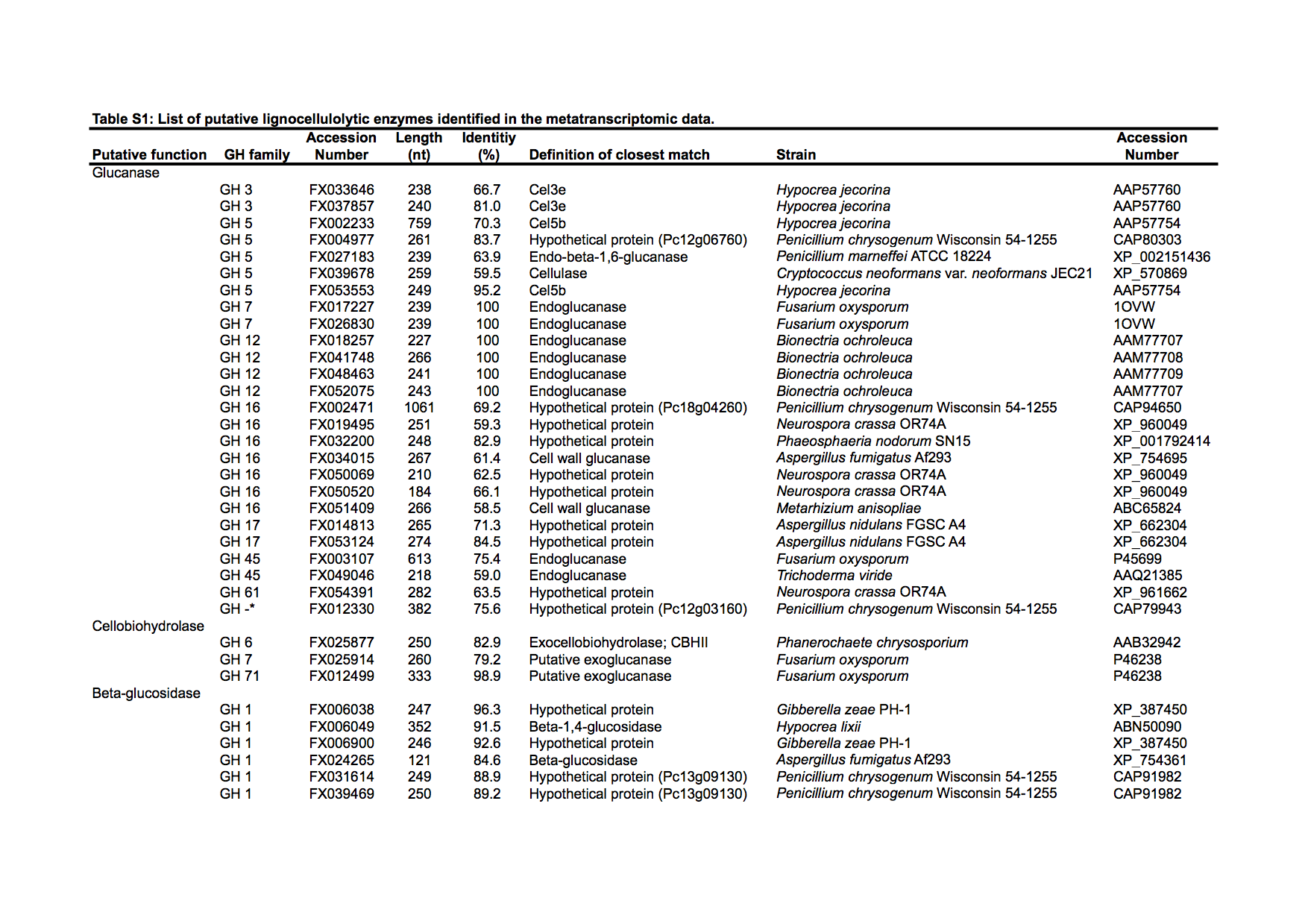

Supplement: Table S1 — List of putative lignocellulolytic enzymes identified in the metatranscriptomic data. (TIFF) [file pone.0055485.s003.tiff]
